# Similar Biomechanical Behavior in Gait Analysis between Ceramic-on-Ceramic and Ceramic-on-XLPE Total Hip Arthroplasties

**DOI:** 10.3390/life11121366

**Published:** 2021-12-08

**Authors:** Athanasios Triantafyllou, Georgios Papagiannis, Vasileios S. Nikolaou, Panayiotis J. Papagelopoulos, George C. Babis

**Affiliations:** 1 Orthopaedic Research and Education Center “P.N.Soucacos”, Biomechanics and Gait Analysis Laboratory “Sylvia Ioannou”, 1st Department of Orthopaedic Surgery, “Attikon” University General Hospital, Medical School, National and Kapodistrian University of Athens, 12462 Athens, Greece; g.papagiannis@go.uop.gr (G.P.); pjportho@med.uoa.gr (P.J.P.); 2 Laboratory of Neuromuscular and Cardiovascular Study of Motion, Physiotherapy Department, Faculty of Health and Care Sciences, University of West Attica, 12243 Athens, Greece; 3 Physiotherapy Department, University of the Peloponnese, 23100 Sparta, Greece; 4 2nd Department of Orthopaedic Surgery, Konstantopouleio General Hospital, Nea Ionia, Medical School, National and Kapodistrian University of Athens, 14233 Athens, Greece; bniko@med.uoa.gr (V.S.N.); gebab@med.uoa.grl (G.C.B.)

**Keywords:** total hip arthroplasty biomechanics, THA kinematics, THA kinetics, ceramic on ceramic THA loading, ceramic on XLPE wear

## Abstract

In vitro measurements are widely used to implement gait kinematic and kinetic parameters to predict THA wear rate. Clinical tests of materials and designs are crucial to prove the accuracy and validate such measurements. This research aimed to examine the effect of CoC and CoXLPE kinematics and kinetics on wear during gait, the essential functional activity of humans, by comparing in vivo data to in vitro results. Our study hypothesis was that both implants would present the same hip joint kinematics and kinetics during gait. In total, 127 unilateral primary cementless total hip arthroplasties were included in the research. There were no statistically significant differences observed at mean peak abduction, flexion, and extension moments and THA kinematics between the two groups. THA gait kinematics and kinetics are crucial biomechanical inputs associated with implant wear. In vitro studies report less wear in CoC than CoXLPE when tested in a matched gait kinematic protocol. Our findings confirm that both implants behave identically in terms of kinematics in a clinical environment, thus strengthening CoC advantage in in vitro results. Correlated to all other significant factors that affect THA wear, it could address in a complete prism the wear on CoC and CoXLPE.

## 1. Introduction

Total hip arthroplasties (THAs) are considered an extremely successful orthopedic operation that provides excellent clinical results and patient satisfaction, followed by long-term survivorship. The British National Joint Registry recently mentioned that THA longevity is 93.2% at 13 years. According to the same data, 80% of these implants show a survival rate of up to 20 years [1,2].

The wear of polyethylene was deemed the weak link of total hip arthroplasty. During the last decade, significant effort has been put toward optimizing wear characteristics and introducing alternative bearings [3,4,5]. After many years of research, the development of highly cross-linked polyethylene (XLPE) with excellent wear characteristics has been a game-changer worldwide. Ceramic femoral heads represent the current trend across many registries [6]. 

In vitro experiments simulating gait kinematics and kinetics [7] have shown the advantages of ceramic-on-ceramic (CoC) bearing surfaces when tested against ceramic-on-polyethylene (CoP) implants. CoC arthroplasties present better osteolytic potential and wear rates [8]; however, their main disadvantage is their increased fragility [9]. In vitro testing of total hip implant designs using constant and variable amplitude loading in a strain-controlled environment along with fracture topography measurements could provide valuable data for their fatigue life predictions [10]. Another critical issue under consideration is that hard-on-hard (HoH) joints could transit higher forces from the prosthesis to the underlying bone due to the absence of the dampening effect of a soft material [11].

Several factors are associated with wear [12], such as implant design variables (geometric properties and material characteristics) [13,14,15,16,17,18], surgical technique variables (implantation approach and positioning) [19,20], and patient-related factors [21,22]. In the latter, the hip contact force, determined by human motion, significantly affects polyethylene wear [23,24]. The sagittal plane’s hip moments explain the wear rate between 42% and 60%, directly associating gait kinematic pattern to implant wear [18]. Although clinical experiments directly correlate gait kinematic pattern with wear of polyethylene in total knee arthroplasties [25], to our knowledge, no in vivo research has examined THA kinematics and its affection to implants’ wear.

The importance of gait biomechanics of THAs on wear, demonstrated by in vitro experiments that simulate gait kinematics and kinetics [26], has been widely used in the last decades to predict wear in THAs. Laboratory trials are an essential component of THA-bearing research. Clinical tests of materials and designs are crucial to prove that accuracy and represent an accurate test validation measure [11].

As defined by the ISO [26], THA wear refers to material loss from the prosthetic joint components due to combined movement and loading and is affected by several factors. One of the most important is gait joint kinematics and kinetics. This is why gait kinematics and kinetics are used in “in vitro” testing protocols, widely accepted methods to predict THA wear. A question raised from such laboratory tests is whether such results show repeatability in clinical measures. In case of mismatching in vitro predictions of wear, new materials or designs should be under consideration [11].

A fact is that all implants are tested with the same kinematic protocol [26] in vitro, and as mentioned above, CoC shows advantages on wear rates compared to CoXLPE in these tests. A vital question arising is, “Do the examined implants offer the same biomechanical behavior in clinical practice too?” 

Our study aimed to examine whether in vitro gait biomechanical data (kinetic parameters and THA kinematics) were replicated during patients’ gait, the essential functional activity of humans, to provide evidence of their contribution to implant wear. These data will be valuable when combined with all the factors mentioned above that affect THA wear to address in a more detailed and complete manner the CoC and CoXLPE wear, the most widely used implant designs. Our study hypothesis was that both implants would present the same hip joint kinematics and kinetics during gait, thus confirming the above-mentioned in vitro results also in vivo.

## 2. Materials and Methods

**Figure 1:**
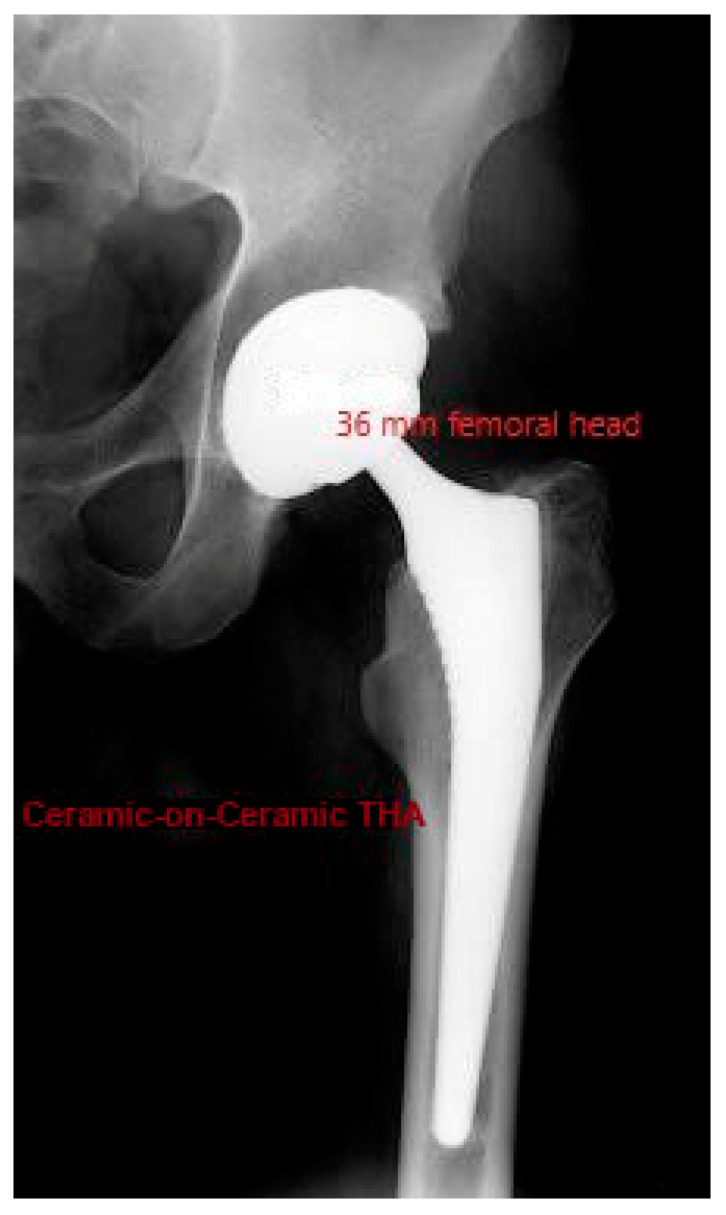
X-ray of ceramic-on-ceramic total hip arthroplasty. A 36 mm femoral head implant was used.

The current study was an investigator-blinded study aiming to find potential differences in peak moments and/or overall ROMs during gait, between 2 groups of patients having unilateral cementless THA of the same make but with different bearing surfaces—36 mm femoral heads, CoC (Figure 1) (Group A) vs. CoXLPE (Group B). The biomechanical variables considered were the total ROMs in sagittal and coronal planes and the highest flexion, extension, and abduction moments (peak moments). To participate in the study, all patients gave informed consent.

### 2.1. Subjects—Clinical Examination

From 2012 to 2016, the same orthopedic surgeon performed 257 consecutive unilateral primary cementless total hip arthroplasties through a minimal posterolateral approach. A total of 130 patients were excluded from gait analysis focusing on the operated hip for any reason that the literature refers that could affect gait pattern: 17 patients who were still using walking aids, 4 patients due to ipsilateral TKA, 8 patients with Parkinson’s disease, 4 patients due to fracture not related to the arthroplasty, 31 due to other illnesses or comorbidities that caused gait deficits or locomotion difficulties (pre-operative gait abnormalities, leg length discrepancy, spine pathologies, dementia, etc.), and 1 due to contralateral hip arthroscopy. In addition, 9 patients had passed away, and 39 patients refused to undergo gait analysis. From the remaining 127 patients, 68 hips had CoC (Group A), and 59 hips had CoXLPE (Group B) bearing surfaces. In all cases, a Pinnacle^®^ cup and a Summit^®^ femoral stem were utilized, DePuy Synthes. The ceramic inserts were Ceramax, Biolox^®^ Delta DePuy Synthes, and all femoral heads were Biolox^®^ Delta DePuy Synthes. The mean time after surgery was 3.2 years for CoC (from 2.8 to 4.2 years) and 2.9 years for CoXLPE (from 2.5 to 4.4 years), respectively, *p* = 0.174. Group A had an average age = 71.2 years (SD = 1.99), and Group B had an average age = 69.3 years (SD = 1.78). This postoperative time was considered sufficient for all patients to adopt their gait pattern of convenience. 

According to the Oxford Hip Score (OHS), the clinical examination showed no significant differences between 2 groups. OHS mean values were 39 (SD = 4.43) for Group A and 37 (SD = 5.01) for Group B, respectively (*p*-value = 0.072). All OHS results were collected during the same session as the gait measurement.

The tools used for collecting the anthropometric characteristics were a Seca (803 model) scale for the body mass and height measurement for the body height. Following pelvic depth, the distance between the anterior superior iliac spine (ASIS) and the knee and ankle joint’s diameters were measured with a caliper. 

### 2.2. Hip Range of Motion Measurements

All subjects’ overall hip joint ROMs at the sagittal, coronal, and axial planes were measured before collecting biomechanical measurements (gait) [27,28,29,30]. 

When hip flexion ROM was measured, the subjects’ position was supine with the knees and hips in neutral rotation. They were asked to flex their operated leg toward their stomach. The goniometer’s fulcrum was set at the femoral greater trochanter, the stationary arm parallel to the trunk, and the movement arm parallel to the femur’s longitudinal axis, aligned with the lateral femoral condyle.

For the acquisition of extension ROM, patients were in the prone position, the goniometer set in the same place as for the flexion movement measurement, and they were instructed to lift their leg straight, as high as possible. 

Measurements for abduction–adduction ROM were carried out with patients lying supine on the testing table. So the knees and the hips were placed in the neutral position, and the fulcrum was placed over the ASIS on the measured side, the stationary arm directed to the contralateral ASIS, and the movement arm parallel to the femur lined up with the patella’s center. The patients were then instructed to bring their leg out to measure abduction and in for adduction, avoiding any rotation of the measured leg or pelvis. 

Hip joint ROMs for internal and external rotation were evaluated with patients sitting on the test table. The hip and knee joint was placed to 90 degrees of flexion. The fulcrum was placed in the center of the patella, with the stationary arm perpendicular to the floor and the movement arm parallel to the tibia’s long axis. The patients were then asked to bring their feet out, and the clinician measured the internal rotation and the external rotation, respectively. 

The mean flexion was 113 degrees (SD = 4.655) and 115 degrees (SD = 4.447) for Groups A and B, respectively, *p*-value = 0.108. The mean abduction was 31 degrees (SD = 4.36) and 30 degrees (SD = 4.487) for Groups A and B, respectively, *p*-value = 0.193, whereas the mean adduction for Group A was 20 degrees (SD = 3.214) and for Group B, 19 degrees (SD = 2.678), *p*-value = 0.181.

Very similar scores between the two groups were noted regarding hip internal rotation ROM. For Group A, the mean value of internal rotation was 36 degrees (SD = 2.91), and for Group B, 35 degrees (SD = 3), with *p*-value= 0.255. No statistically significant differences were found for the scores (mean values) of external hip rotation between two groups (Group A 36 degrees (SD = 3.181), Group B 34 (SD = 2.815), and *p*-value = 0.317).

Following the clinical examination results for hip joint ROMs and OHS, as mentioned above, the subjects of both groups were considered capable of instrumented gait analysis. 

These groups were comparable in gender, age, the reason for THA, type of implant, Oxford Hip Score, and ROM of the operated hip. In addition, the biomechanical data were normalized to body weight and height. 

### 2.3. Gait Analysis

Two force plates (Kistler) were placed on the floor and integrated with an eight-optoelectronic-camera BTS motion analysis system and were used to measure the forces applied on the hip. The issue of measurements’ accuracy was approached with the system’s standard calibration before acquisition. This procedure was also necessary to accurately compute three-dimensional (3D) marker coordinates during the trials. The same clinician-biomechanist carried out the whole process. The measured and actual distance, i.e., mean difference, concerning 2 markers that were fixed on the segment of a rigid bar at a distance of 0.6 m was within 0.003 m. The calibrated volume of the laboratory space was 10 m × 3 m in the sagittal plane (x-axis), 3 m × 3 m in the coronal plane (y-axis), and 3 m × 10 m in the axial plane (z-axis). All axes and planes refer to the laboratory reference system. 

A file was created and saved at the end of each session, including calibration records and acquisition data. 

The technical features of the force plates were:

➢ Crosstalk less than 1%;
➢ Linearity less than 0.5% of full scale;
➢ Hysteresis less than 0.5% of full scale.

The frequency of the 8 optoelectronic cameras and force platforms was set to 120 Hz to measure gait activities, as suggested by the literature. 

As for the accuracy of the motion analysis system, its error was less than 0.001 m for the calibrated laboratory space mentioned above (10 m × 3 m × 3 m). Finally, the calibrated range of the force plates was set from 0 to 10,000 N to measure the maximum subject’s mass of 1000 N. These setting and calibration ranges of the gait analysis system ensured the accuracy of input captured during gait analysis [31,32].

Following the setting and system calibration, the biomechanist applied twenty-two markers on each patient, using Davis protocol for the gait analysis of THAs [33]. 

The protocol followed to ensure the reproducibility of the measurements included a static trial from the body’s anatomical position. According to the literature [34] for gait analysis methodology, the statical trial is the procedure used to minimize possible errors regarding three-dimensional motion capture when skin markers (external) are used. This procedure ensures that all body segments can be correctly and fully reconstructed from the system during gait acquisition and permitted any corrections needed to be made because of markers’ misalignment before dynamic trials. Furthermore, each joint’s definition of inaugural degrees was captured and used to reference the segmental movements in all planes during gait. 

For the static trial, the subjects were instructed to the proper human body anatomic position. Then they were asked to place both legs on 1 of the 2 force plates, with their feet parallel at a distance of 0.15 m, and stay still. 

Before the dynamic trial, instructions were given to all patients for the upcoming gait acquisition to avoid targeting the force platforms by using “trick” steps and rest periods applied between gait trials to prevent fatigue. All dynamic trials were controlled for the effect of fatigue or distraction to ensure that no unrepresentative data were passed at the next statistical analysis stage. The criterion for the collection of representative data was normal gait style and speed. In all cases, 3 gait cycles were performed to ensure the measurements’ repeatability [35]. 

Finally, before the analysis, all collected data were checked for marker’s trajectory continuity, clear force platforms strikes, and absence of “trick” steps [36]. Random sample curves of joint ROMs and moments were examined before the patient left the laboratory. The same clinician, Ph.D. in biomechanics, completed the measurement, calibration, and data collection procedure, being blind to the type of articulation. Informed consent was obtained from all participants, and their rights were fully protected.

### 2.4. Data Analysis 

Anthropometric measurements were combined with three-dimensional marker data from the static trial to define the joint’s center and anatomic axes of hip joint rotation. The position of the markers provided the three-dimensional joint angles. Combined with the external forces, these data allowed the calculation of corresponding moments [37]. All data, including marker identification, angular displacement, and joint moment calculations, were conducted using BTS Smart Clinic^®^ software ver. 1.10.470.0. Kinetic forces produced from the musculoskeletal modeling and force plates were normalized to body weight and moments to the percentage of body weight and height. External moments were calculated using inverse dynamics and amplitude normalized to body mass (Newton meter per kilogram) [38].

### 2.5. Statistical Analysis

Independent t-tests were used to compare differences in Oxford Hip Scores and hip ROMs between the two groups. The same statistical tool was used to determine whether there was a statistically significant difference in kinetic and kinematic data extracted from gait analysis comparing CoC and CoXLPE.

## 3. Results

### 3.1. Kinetic Data

#### 3.1.1. Peak Abduction Moment

**Figure 2:**
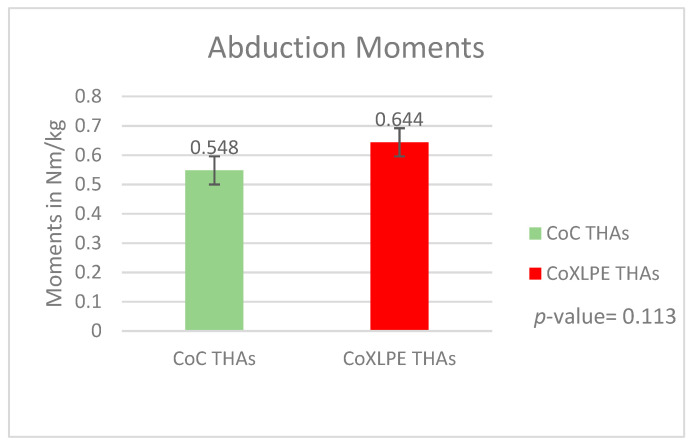
Abduction moments during gait.

The mean peak abduction moment was 0.548 Nm/kg (SD = 0.012) and 0.644 Nm/kg (SD = 0.015) in CoC (Group A) and CoXLPE (Group B) THAs, respectively (*p*-value = 0.113). These peak values were produced at 18.27 percent of the gait cycle for CoC (SD = 4.789) and at 22.5 percent of the gait cycle for the CoXLPE group (SD = 4.857) (*p*-value = 0.125). The graphs of the mean peak values for both groups are presented in Figure 2.

#### 3.1.2. Peak Extension Moments

**Figure 3:**
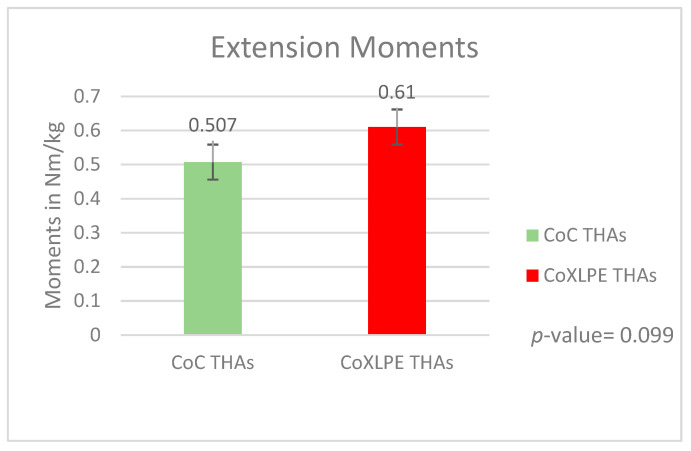
Extension moments during gait.

The mean peak extension moment was 0.507 Nm/kg (SD = 0.014) and 0.61 Nm/kg (SD = 0.015) in CoC and CoXLPE THAs, respectively (*p*-value = 0.099). These peak values were produced at 11.22 percent of the gait cycle for CoC (SD = 2.432) and at 13.05 percent of the gait cycle for the CoXLPE group (SD = 2.414) (*p*-value = 0.082). The graphs of the mean peak values for both groups are presented in Figure 3.

#### 3.1.3. Peak Flexion Moments

**Figure 4:**
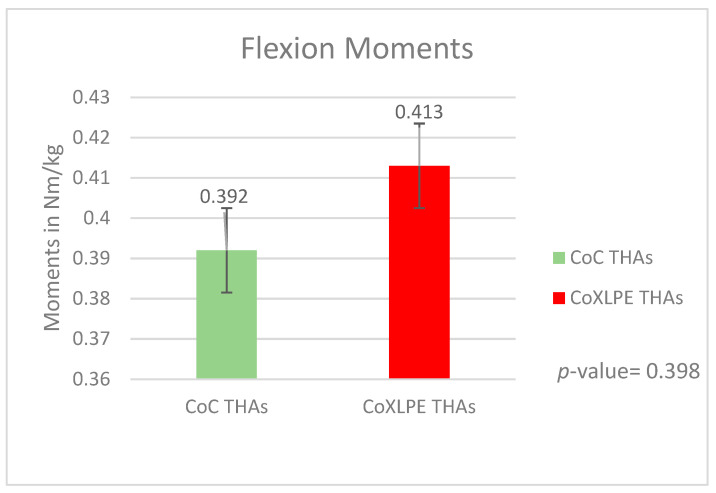
Flexion moments during gait.

The mean peak flexion moment was 0.392 Nm/kg (SD = 0.013) and 0.413 Nm/kg (SD = 0.016) in CoC and CoXLPE THAs, respectively (*p*-value = 0.398). These peak values were produced at 54.49 percent of the gait cycle for CoC (SD = 5.693) and at 56.38 percent of the gait cycle for the CoXLPE group (SD = 4.584) (*p*-value = 0.218). The graphs of the mean peak values for both groups are presented in Figure 4.

### 3.2. Kinematic Data

#### 3.2.1. Flexion–Extension ROMs

**Figure 5:**
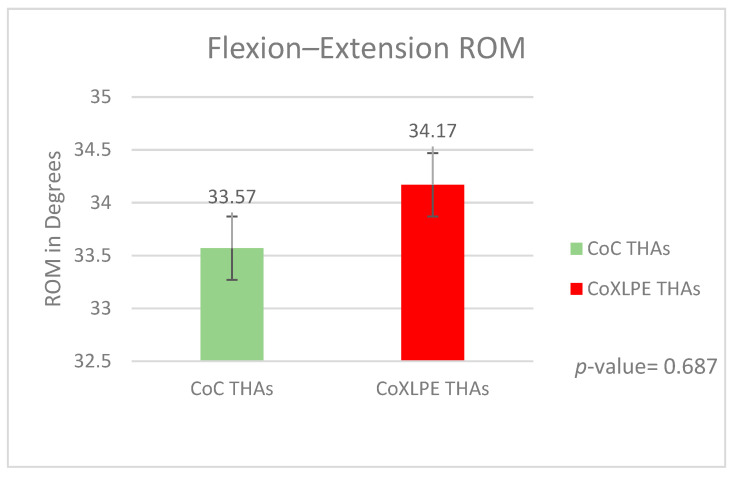
Flexion–extension ROMs during gait.

According to the calibration protocol and Cartesian coordinates, positive values in sagittal plane ROMs correspond to hip flexion, while negative values refer to extension. The total ROM was calculated by summing the absolute maximum values of flexion and extension angles. The mean total ROM was 33.57 degrees (SD = 3.76) and 34.17 degrees (SD = 2.79) for Groups A and B, respectively, *p*-value= 0.687 (Figure 5).

#### 3.2.2. Abduction–Adduction ROMs

**Figure 6:**
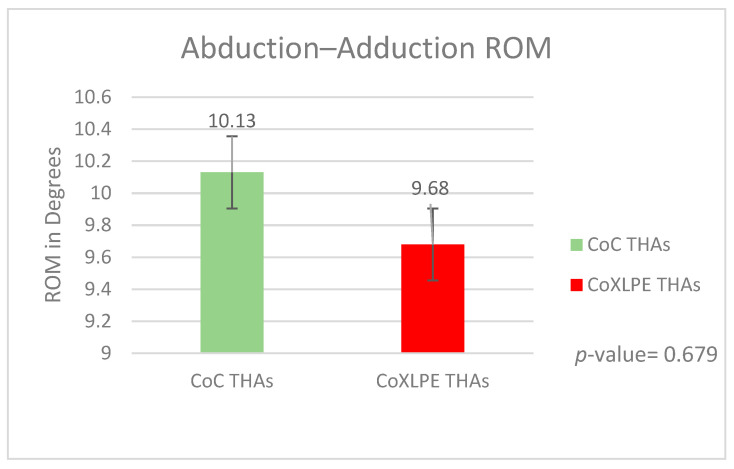
Abduction–adduction ROMs during gait.

Similarly, for the coronal plane, positive values correspond to adduction angles and negative to abduction angles. The total ROM value for statistical analysis was calculated by summing the absolute maximum abduction and adduction angles. The mean total ROM was 10.13 degrees (SD = 2.22) and 9.68 degrees (SD = 2.14) for Groups A and B, respectively, *p*-value = 0.679 (Figure 6).

## 4. Discussion

Our study assumed that both implants would present the same hip joint kinematics and kinetics during gait. Our results show minor differences in peak abduction and extension torques between these two groups, with a slightly higher mean peak abduction and extension moment in the CoXLPE THA group (not statistically significant, *p*-value = 0.113 for abduction, *p*-value = 0.099 for extension, and *p*-value = 0.398 for flexion peak moments). The kinetic data in our study showed excellent repeatability for both groups. The mean peak values of hip flexion, extension, and abduction moments appeared in identical time instants of the % gait cycle. In conjunction with no differences in peak moments, that finding suggests no differences in the kinetic behavior either at the sagittal or coronal planes during gait. 

No statistically significant differences were identified regarding the overall flexion–extension (*p*-value = 0.687) and abduction–adduction (*p*-value = 0.679) ROMs between both groups during gait analysis. Therefore, we conclude that the two bearing types show similar kinematic behavior.

Laboratory testing of hip prosthesis wear involves a standard simulated walking cycle [39]. In vitro studies [26] report less wear in CoC than CoXLPE when tested with the same gait cycle kinematic protocol. The main issue of these in vitro measurements is that if their results/predictions are not replicated when testing in a clinical environment, a question is raised regarding the material tested or the implant design. An example is the ultra-high molecular weight polyethylene (UHMWPE) Hylamer^®^. In this case, the published hip simulator in vitro testing did not prospectively predict in vivo behavior [11]. Our findings show that both implants’ kinematic pattern is identical in clinical practice too. This result confirms that both implants behave identically in terms of kinematics in a clinical environment. Since the research hypothesis was verified, our study’s most important finding is that CoC should have a wear advantage compared to CoXLPE when gait kinematics are studied. This advantage should be correlated to the rest of the factors affecting wear on THAs to answer the question of which of the two examined materials has an “overall” better wear behavior in clinical practice.

Optoelectronic cameras combined with force plates are the gold standard technique for three-dimensional gait analysis and measurements of the ROM and moments applied on lower extremities’ joints [39,40]. Gait is the essential functional activity of humans. Under its particular conditions, THAs are subject to loading; thus, such kinematic and kinetic data are of great biomechanical importance [41,42].

The fact that gait directly affects total hip arthroplasties’ wear and tear is well studied and clearly defined [36,42]. Davey et al.’s research results state that the gait pattern of patients subjected to THA with the use of ultra-high molecular weight polyethylene (UHMWPE) acetabular component was an essential factor influencing prosthesis wear and biocompatible effectiveness in long-term use [27]. Another study evaluated the efficacy of total hip arthroplasty one year postoperatively [26]. The results mention that postoperative gait improvement significantly increased the hip’s load capacity and reduced prosthesis loosening risk. Since both implants’ bearing surfaces show the same kinetic behavior during gait, we assume that the same peak joint loading does not differ in kinetic hip function. Similarly, hip joint moments do not provide evidence for discrepancies in wear rates in any examined THAs.

Recent findings suggest that both high molecular weight polyethylene (HMWP) [9] and XLPE [43], as a result of loading, could generate both smaller and more biologically active wear debris. As a result of this behavior, these bearings could show increased wear rates. On the other hand, HoH implant designs such as MoM (metal on metal) [44] and CoC [45] bearings became popular in the effort to deal with the possible risk of osteolysis induced by polyethylene wear debris as they present lower wear rates than polyethylene [46]. Alumina ceramic debris is less biologically active than the highly cross-linked polyethylene one [47]. The considerable clinical alteration in the wear volumes noticed when coupled with polyethylene acetabular cups [8] may be correlated with the level of activity and patients’ weight [9], the composition of the lubricant [6,11], the presence of third-body damage [8], and the oxidative state of the polyethylene [48]. However, recent in vivo fluoroscopic studies have revealed some interesting differences in kinematic patterns [49]. Specifically, the literature reports a micro separation of THA head and acetabular components between toe-off and heel strike gait phases [26]. This phenomenon is reported to be higher when polyethylene cups are articulated to ceramic or metal heads than HoH articulations. The micro separation affects the lever arm of the externally applied forces, affecting the hip torques too. 

### 4.1. Proposition for Further Work

Our study concluded that none of the two THAs is at a biomechanical advantage in kinematics or kinetics during gait. However, our findings should be used in further research where computational biomechanical modeling could predict the actual force distribution between the articulated components of the examined THAs. 

This finding should also be taken into account when examining the influence of wear on THA longevity. In addition, it should be correlated with the rest of the risk factors to evaluate the implants’ longevity more accurately. 

Nowadays, sensor technology (step activity monitor (SAM), Cymatech, Seattle, Washington, PDMonitor) can accurately measure the number of steps, ROMs, and kinematic patterns during the gait of patients subjected to THA. Such kinematic information, combined with our findings (kinematic and kinetic data from gait analysis) and surface electromyography (sEMG) gait analysis data [50,51,52], could help develop more precise protocols of in vitro testing to predict the wear rate of implants accurately. 

### 4.2. Sources of Errors

Possible sources of errors refer to the fact that when external skin markers are applied to the human body for motion analysis, artifacts are produced by the skin movement. Such incorrect movement estimations are sometimes present during the swing phase (which was not a phase of interest in our study). On the other hand, the point cluster method followed during gait analysis can sufficiently reduce these artifacts. In conclusion, the literature clearly states that when gait methodology is tracked accurately and adequately, such errors cannot influence the data collection when kinetic and kinematic parameters are being measured using optoelectronic cameras and piezoelectric force plates.

## 5. Conclusions

In our study, the peak values of the examined abduction, adduction, and flexion moments (*p*-values 0.113, 0.099, and 0.398, respectively) and the overall flexion–extension and abduction–adduction ROMs (*p*-values 0.687 and 0.679, respectively) presented no statistically significant differences. These similar results of kinematic and kinetic gait analysis data in hard-on-soft vs. hard-on-hard bearing surfaces when correlated with in vitro tests suggest that CoC kinematic behavior could show a comparative advantage on wear rates to CoXLPE THAs. This advantage should be correlated with the rest of the factors affecting wear on THAs to answer the question of which of the two examined materials has an “overall” better wear behavior in clinical practice.

## Data Availability

Data available on request due to restrictions of privacy.

## Abbreviations

THA	total hip arthroplasty
CoC	ceramic on ceramic
CoXLPE	ceramic on highly cross-linked polyethylene
CoP	ceramic on polyethylene
HoH	hard on hard
ISO	International Standardization Organization
ROM	range of motion
SD	standard deviation
OHS	Oxford Hip Score
ASIS	anterior superior iliac spine
3D	three-dimensional
UHMWPE	ultra-high molecular weight polyethylene
HMWP	high molecular weight polyethylene
SAM	step activity monitor
sEMG	surface electromyography
